# Diversity of Phenotype and Genetic Etiology of 23 Cystinuria Saudi Patients: A Retrospective Study

**DOI:** 10.3389/fped.2020.569389

**Published:** 2020-11-11

**Authors:** Malak Alghamdi, Khalid A. Alhasan, Areej Taha Elawad, Suha Salim, Marwa Abdelhakim, Marwan Nashabat, Rupesh Raina, Jameela Kari, Majid Alfadhel

**Affiliations:** ^1^Medical Genetics Division, Department of Pediatrics, College of Medicine, King Saud University, Riyadh, Saudi Arabia; ^2^Nephrology Division, Department of Pediatrics, College of Medicine, King Saud University, Riyadh, Saudi Arabia; ^3^Computer, Electrical & Mathematical Science and Engineering Division (CEMSE), Computational Bioscience Research Center (CBRC), King Abdullah University of Science and Technology (KAUST), Thuwal, Saudi Arabia; ^4^King Abdullah International Medical Research Center, King Saud bin Abdulaziz University for Health Sciences, King Abdulaziz Medical City, Ministry of National Guard-Health Affairs, Riyadh, Saudi Arabia; ^5^Department of Nephrology, Cleveland Clinic Akron General, Akron, OH, United States; ^6^Pediatric Nephrology Center of Excellence and Department of Pediatrics, College of Medicine, King Abdulaziz University, Jeddah, Saudi Arabia; ^7^Division of Genetics, Department of Pediatrics, King Abdullah Specialized Children Hospital, King Abdulaziz Medical City, Ministry of National Guard-Health Affairs, Riyadh, Saudi Arabia

**Keywords:** cystinuria, SLC3A1, inborn errors of metabolism, SLC7A9, nephrolithiasis, dibasic amino acids

## Abstract

**Background:** Cystinuria is an inborn error of metabolism that manifests with renal stones due to defective renal epithelial cell transport of cystine which resulted from pathogenic variants in the *SLC3A1* and/or *SLC7A9* genes. Among nephrolithiasis diseases, cystinuria is potentially treatable, and further stone formation may be preventable. We report 23 patients who were identified biochemically and genetically to have cystinuria showing the diversity of the phenotype of cystinuria and expanding the genotype by identifying a broad spectrum of mutations.

**Patients and Methods:** This is a multicenter retrospective chart review, where clinical and biochemical data, genetic analysis and the progress of the disease were documented over five years at two centers from 2014 to 2019.

**Results:** Of 23 patients who were identified biochemically and/or genetically to have cystinuria, 14 (62%) were male. Thirteen patients were homozygous, and two were heterozygous for the *SLC3A1* gene. Seven were homozygous and one was compound heterozygous for the *SLC7A9* gene. We have detected 12 genetic variants including five novel variants. *SLC3A*1 gene variant c.1400 T > A (p.Met467Lys) is found in 38% of our cohort. Although 21 patients required surgical intervention, none developed ESRD. The number of stone episodes per year varied widely (median frequency of 0.45 stones/ per year, range between 0.06 and 78.2), with no significant difference in stone events per year between sexes (*P* = 0.73).

**Conclusion:** Despite the high rate of consanguinity in Saudi Arabia, there was a broad spectrum of genetic variants. Most of our patients are homozygous recessive for *SLC* genes with multiple generations affected which indicates early screening and prevention of disease in these families. Phenotypic heterogeneity is well documented in our cohort even with the same genotype and the first stone episode age was variable but most commonly seen in the first decade of life.

## Introduction

**Figure 1:**
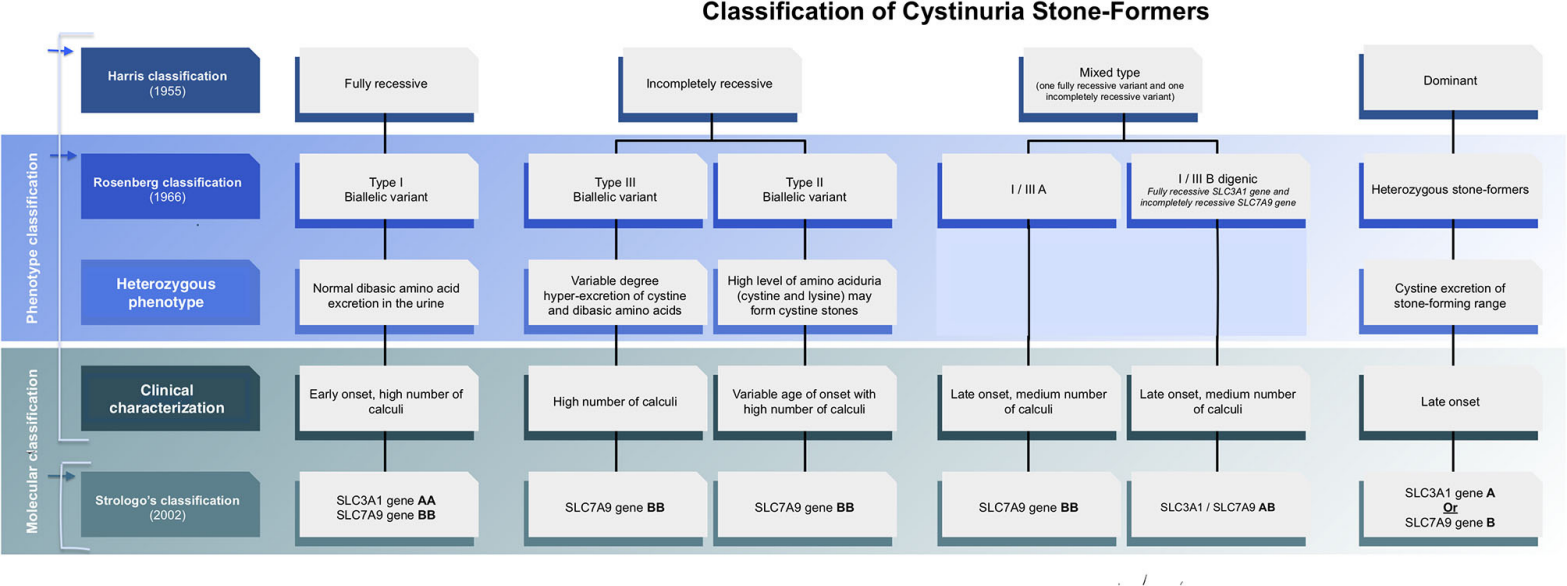
Evolving classification of cystinuria stone formers.

Cystinuria is an inherited metabolic disorder affecting the dibasic amino acid transporter in the proximal convoluted tubule of the kidneys (1, 2). It is characterized by inadequate reabsorption of cystine and dibasic amino acids in the kidney that results in excessive urine excretion of cystine and the dibasic amino acids lysine, arginine, and ornithine due to defective transepithelial transport of these amino acids in the proximal tubule and the small intestine. The genes responsible for the reabsorption of cystine and dibasic amino acids in the kidney are *SLC3A1* or *SLC7A9*, which encode the two subunits of the amino acid transport system b(0,+). *SLC3A1* encodes the type II membrane glycoprotein (rBAT), which transports neutral and basic amino acids in the renal tubule and intestinal tract. The high affinity and sodium-independent transport of cystine and neutral and dibasic amino acids is the role of the protein [b(0,+)-type amino acid transporter 1] that is encoded by *SLC7A9* (Figure 1). This results in recurrent cystine renal stones, obstructive uropathy, hypertension, infection, and, rarely, renal failure or progressive renal disease (3). Patients may also require multiple surgical interventions to remove stones. Given the morbidity associated with cystinuria, it is considered one of the most challenging nephrolithiasis diseases to treat (2, 4).

Classification of cystinuria can be done clinically, biochemically, or genetically. According to gene-based classification, type A and type B cystinuria are associated with mutations of both alleles of *SLC3A1* or *SLC7A9*, respectively (5) (Figure 1). Cystinuria caused by mutations in *SLC3A1* (Type A) is an autosomal recessive condition. Previous studies suggest that patients who are heterozygous for mutations in *SLC3A1* have normal urinary cystine and dibasic amino acid levels (6, 7), but it has been shown later that heterozygotes individuals also might have elevated levels of urinary cystine (8) and can develop recurrent cystine stones which indicate dominant pattern (6, 9). The inheritance of *SLC7A9* mutations (Type B) is usually autosomal dominant with variable penetrance, with 85% of *SLC7A9* heterozygotes having abnormal urinary dibasic amino acid levels (7), some of whom develop cystine stones. Later reports showed the *SLC7A9 gene* recessive pattern as well. In summary, the inheritance pattern of cystinuria is complex, classical recessive patterns have been proven and classical dominant patterns also have been demonstrated but in between uncertain patterns due to digenic inheritance and reduced penetrance phenomena (10–12). Indeed, molecular with biochemical studies are required to be able to determine the inheritance pattern and screen the family members at risk.

The diagnosis of cystinuria is made by the detection of cystine stones and the measurement of excreted cystine and dibasic amino acids in the urine. Renal ultrasound imaging is the method of choice for stone detection and follow-up as well. Genetics testing is used to confirm the diagnosis molecularly and for family counseling. There are no diagnostic or therapeutic indications for performing genetic testing. However, genotyping might direct the clinicians and the family to relevant family members who required screening.

Treating cystinuria aims to reduce cystine in urine. Hydration to dilute the urine, urinary alkalization to increase cystine solubility (with potassium citrate), and administration of cystine-binding medications (α-mercaptopropionylglycine and D-penicillamine) to produce more soluble cysteine-drug product and lower free cystine levels in the urine are all viable therapies. When the cystine stone is small (under 12 mm), extracorporeal shock wave lithotripsy (ESWL) is used, but it is reported to have low efficiency due to the consistency of cystine stones. For large stones, laser stone fragmentationor percutaneous nephrolithotomy is necessary (2).

This study highlights the diversity of cystinuria genotype and phenotype in the Saudi population as well as describes the course of the disease over 6 years. A retrospective chart review of 23 cystinuria patients from 18 unrelated families was carried out. Patients were diagnosed and treated at King Khaled University Hospital and King Abdullah Specialized Children's Hospital in Riyadh, Saudi Arabia, creating the largest cohort of patients with cystinuria in Saudi Arabia. We describe the genetic variations/genotype identified and clinical courses/phenotypes of these patients to personalize cystinuria management in Saudi Arabia.

## Methods

### Clinical Analysis

All patients in this study had a diagnosis of cystinuria on the basis of confirmed cystine stone(s) on chemical analysis or biochemical and/or molecular studies. Detailed clinical data were collected retrospectively from the Nephrology and Medical Genetics Clinics at King Saud University Medical City and King Abdullah Specialized Children's Hospital from 2014 to 2019. The data include results of patients' genetic analysis, clinical onset, and progress of the disease was documented over a period of 5 years. The assigned physicians completed data collection for each patient under their care, including age, gender, ethnicity, family history, growth parameters, blood pressure, age at diagnosis, age at first stone presentation, and number of surgical interventions for stone removal.

### Biochemical Analysis

We performed relevant blood and urine biochemistry, including urine amino acid (mmol/mmol of creatinine), plasma amino acids, stone analysis, renal function tests, and genetic tests, as well as detailed renal ultrasound studies.

### Genetic Study

Genomic DNA was enzymatically fragmented, and regions of interest were selectively enriched using capture probes targeted against the coding regions of selected genes. Libraries were generated with Illumina-compatible adaptors and sequenced on an Illumina platform.

The evaluation focused on coding exons along with the flanking ±10 intronic bases of the *SLC3A1* and *SLC7A9* genes. Raw sequence data analysis, including base calling, demultiplexing, alignment to the hg19 human reference genome (Genome Reference Consortium GRCh37), and variant calling [single nucleotide variants, indels and copy number variations (CNVs)] was performed using validated in-house software. Relevant variants reported in HGMD and ClinVar, as well as all variants with a minor allele frequency (MAF) of <1% in the genome AD database, were considered. All pertinent inheritance patterns were considered. In addition, provided family history and clinical information were used to evaluate eventually identified variants. The reference laboratory has established stringent quality criteria and validation processes for variants detected by next-generation sequencing (NGS). Lower quality single nucleotide or deletion insertion variants were thus confirmed by Sanger. As a result, we required a specificity of >99.9% for all reported options.

Two independent CNV callers were used to determine CNVs within the panel genes from the NGS data. While one caller used the Bayesian approach to distinguish biological from technical differences for each target region, the second used a mixture of Poisson models and compared the coverage of small fragments within the target regions to a set of reference samples. Implementation of the two callers in tandem offers two alternative approaches to detect CNVs, thereby complementing each other for a robust and sensitive CNV calling pipeline. All clinically relevant CNVs were confirmed with an orthogonal method (MLPA, multiplex ligation-dependent probe amplification or qPCR, quantitative polymerase chain reaction) before reporting. MLPA analyses were performed using SALSA MLPA probemix P426-A1 provided by MRC-Holland to test for deletions or duplications within or including the *SLC3A1* or *SLC7A9* gene(s).

## Results

### Clinical Evaluation

In total, 23 patients diagnosed with cystinuria were identified, including 9 (38%) female and 14 (62%) male patients. Six adults had been followed up since early childhood, three females and three males. All patients had their first diagnosis of renal stones made in childhood. Thirty-nine percent of patients in the current cohort presented in the first year of life: 30% between 1 and 5 years, 22% between 5 and 10 years, and 9% after 10 years of age. The median age at first presentation with renal stones was three years (range = 0–20 years). All patients were Saudi except three, with one each from Kuwait, Yemen, and Lebanon. Positive family history of cystinuria was documented in 12 (52%) patients; 7 patients had only one generation affected (siblings), one patient had two generations affected, and 4 (19%) patients had a history of cystinuria in three generations.

Stone formation per year varied widely (median frequency of 0.45 stones per year, ranging between 0.06 and 78.2, with no significant difference in stone formation per year between sexes (*P* = 0.73). We have documented stone recurrence either as symptomatic recurrence (pain and hematuria and/or UTI) which required in ER visits and asymptomatic recurrence detected radiological either by U/S and CT scan during routine clinic follow up (13).

All 23 patients in our study had been advised to increase daily intake of fluids. Additional management, including alkalizing and/or cystine-binding treatments, were prescribed to all patients (Table 1). Twelve patients (52%) received monotherapy, and 11 (48%) patients were given a combination of alkalization and cystine-binding therapies. Of the six patients previously or presently taking tiopronin, 2 patients were noted to have discontinued alkalization medication because they found the potassium citrate unpalatable. Overall, there was evidence that all 21 patients strictly adhered to medical therapy, which followed international guidelines (14, 15). Despite this, 19 (90%) of the patients continued to form recurrent kidney stones while on medical therapy.

**Table 1:** Long-term follow-up with radiological findings to show the progression of the disease while on treatment.

**No**.	**1st scan**		**Age at first scan**	**Intervention**			**2nd scan**		**Age at latest scan**
	**Rt**	**Lt**		**Medical**	**Surgical**		**Rt**	**Lt**	
					**Age at first surgery**	**Intervention**			
1	N	N	3 years	Potassium Citrate	3 years	Surgical removal of bladder stone	N	N	7 years
2	N	N	1 years	Potassium Citrate	1 years	Cystourethroscopy warer scop	Mild pelvic fullness	N	5 years
3	N	N	2 years	Potassium Citrate	2 years	2 times	N	Stone in kidney (1.8 cm)	16 years
4	UL	UL	8 years	Potassium Citrate	8 years	3 times	Bilateral Stone (1.6 cm)	Bilateral stones (0.9 cm)	11 years
5	N	N	2 years	Potassium Citrate	2 years	>4 times	Renal Stones	Renal stones	10 years
6	Renal stone	Renal stone	8 months	Potassium Citrate	8 months	2 times		Unchanged small stones	3 years 8 months
7	UL	UL	1 years	Potassium Citrate	1 years	PCNL	N	N	4 years
8	N	UL	3 years	Potassium Citrate	3 years	None		Small renal stone	4.5 years
9	G2. hydronephrosis	N	2 years	Potassium Citrate/D penicillamine	2 years	Multiple URS and PCNLs > 5	Renal stones	Renal stones	>14 years
10	Horseshoe kidneys		9 years	Potassium Citrate	9 years	PCNLs/4 times	Small stones	Small stones	16 years
11	HN	HN	8 years	Potassium Citrate + Tiopronin	8 years	4 times lithotripsy	Bilateral multiple stones; largest 14 mm	Bilateral stones	13 years
12	N	Small stone with mild HN	6 months months	Potassium Citrate+ Pencillnamin	6 months	PCNL	No stones seen	No stones seen	1 years 6 m
13	Small stone	N	4 years	Potassium Citrate	4 years	None	Multiple Stones	N	10 years
14	N	N	20 years	Potassium Citrate	20 years	PCNL	N	N	21 years
15	Multiple stone	N	8 years	Potassium Citrate	8 years	Rt. nephrectomy	N after nephrectomy	N	22 years
16	Renal stone and HN	Renal stone and HN	4 years	Potassium citrate	11 years	PCNL + ESWL	Interval increase in the size of nonobstructive right renal stones (largest 2.2 x 1.4 x 0.8 cm compared to 1.5 x 1.1 x 0.7 cm.) Interval improvement in bilateral HN	Stable size left renal nonobstructive stones. Interval improvement in bilateral HN	16 years
17	Renal stones in the lower pole	N	11 years	Potassium Citrate	16 years	Rt. nephrectomy PCNL	Surgically removed	Solitary left kidney. No renal stones or hydronephrosis seen.	23 years
18	Stones	N	17 years	Potassium Citrate, Tiopronin	23 years	ESWL PCNL	Renal stone	N	23 years
19	Stones	Nonobstructive stones	3 years	Tiopronin HTN on amlodipine and lisinopril	3 years	PCNL	DMSA scan: There is heterogeneity of tracer uptake in both kidneys. However, there are no well-defined cortical defects to suggest scarring	Abdominal CT: progression increase in the size of the lower pole renal stone staghorn type	11 years
20	N	Multiple stones G1-HN	2 years	Enalapril Potassium Citrate	2 years	PCNL	Stable	Stable	
21	Normal	Nonobstructive, tiny stones	10 years	Lisinopril	12 years	PCNL	Normal	CT scan: Nonobstructive tiny stones in the left kidney.	19 years
22	UL	N	10 years	-	12 years	PCNL	Stable	Multiple lower pole calculi.	13 years
23	Normal	Partially obstructing stone	16 years	Azathioprine for ulcerative colitis.	17 years 20 years	PCNL 2X	Normal	Staghorn stone 4 cm.	20 years

The majority of patients had normal eGFR. No patients developed ESRD, and none required renal transplantation.

Twenty-one patients (90%) underwent surgical intervention, including non-invasive percutaneous nephrolithotomy (PCNL), extracorporeal shock wave lithotripsy (ESWL), endoscopic surgery including ureteroscopy and lithotripsy, and open pyelolithotomy.

Three patients developed hypertension. Two had recurrent urinary tract infections (UTIs). Stone formation at the last renal ultrasound follow-up was bilateral in 5 (19%) patients, unilateral in 11 (47%), and normal study (no stones) in 7 (33%) patients. One patient had a horseshoe kidney, and one patient presented with a large bladder stone with normal kidneys. Unrelated clinical features included delayed puberty in patient no. 12 and ulcerative colitis in patient no. 23 (Table 1).

### Biochemical Analysis

As shown in Table 2, all patients had elevated total urine dibasic amino acid levels (mmol/mmol of creatinine). One patient had isolated cystinuria with normal levels of the other dibasic amino acid. No correlation was found between high excretion of dibasic amino acids and clinical outcomes (Table 2). None of the heterozygous parents/siblings were symptomatic, and thus they did not undergo biochemical profiling.

**Table 2:** Clinical phenotypes including age of onset, course of the disease and outcome of the intervention.

		**Clinical profile**					**Urinary Biochemical profile**						**Genetic result**		**Outcome**		
	**Sex**	**Onset**	**HTN**	**Proteinuria**	**RF**	**UTI**	**Cystine 0.00–130**	**Ornithine 0.00–7.00**	**Lysine 10–103**	**Arginine 0.00–6.00**	**Total 10–246**	**Gene**	**Allele**	**Variant**	**Uni-bi**	**Thiol drugs**	**Uni. nephrectomy**
1	F	3 years	-	-	N	+	224	0.31	10.3	0.02	234	SLC3A1	Homozy	C.592del (P.Ala198glnfs*8),	Bladder		
2	M	1 years	-	_	N	+	261	202	462	522.57	1,448	SLC7A9	Homozy	C.997_1012del (P.Arg333serfs)	uni		
3	M	2 years	-	_	N	+	157	309	511	532	1,509	SLC3A1	Homozy	C.1400t > A (P.Met467lys)	Uni		
4	M	4 months	-	_	N	-	166	210	459	567	1,402	SLC7A9	Com. Heteroz.	c.376g > A (P.Ala126Thr) c.1166C > T (p.Gly105Arg)	bi		
5	F	2 years	-	_	N	-	112	234	479	459	1,284	SLC7A9	Homozy	C.313g > A (P.Gly105arg)	bi		
6	M	8 months	–	_	N	+	96	529.8	1058	1,004	2,688	SLC3A1	Homozy	Duplication (4 copies) encompassing exon 5 to exon 9	bi		
7	M	1 years	-	+_	N	+	199	27.9	218.9	16.9	462	SLC7A9	Homozy	C368 > T (p.Thr123met)	bi		
8	F	3 years	-	_	N	+	100	167	670	585	1,522	SLC3A1	Homozy	c.1617+1097T > A	uni		
9	M	4 months	-	_	N	+	31.66	78.66	186.32	218	513	SLC3A1	Homozy	C.1400 > A (p.Met467lys)	bi	+ (Pen)	
10	M	9 years	-	_	N	-	65	95.45	256.04	250.2	666	SLC3A1	Homozy	C.1400 > A (p.Met467lys)	bi		
11	M	8 years	-	+	N	+	61.77	156.51	419.87	285	921	SLC7A9	Homozy	C.460_471del (P.Leu154_Ala157del)	bi	+ (Tio)	
12	M	6 months	-	_	N	-	Positive	350	1.162	434	>1,946	SLC7A9	Homozy	1060g > A p.A354t	uni	+ (Pen)	
13	M		-	_	N		266	206,5	539.99	353,19	1,364	SLC3A1	Heterozy.	C.1229a > T (P.Asn410ile) Exon 7	uni		
14	F	20 years	-	_	N	-	65.2	87.6	231	206.5	589	SLC3A1	Homozy.	Variant C.1400>A (p.Met467lys)	bi		
15	M	8 years	-	_	N	-						SLC3A1	Heterozy	c.1229A > T (p.Asn410Ile) Exon 7	bi		+
16	F	Birth	-	N	N	+	160	177	1179	790	2,306						
17	M	Birth	-	N	N	-	310	508	772	3,885	5,325	SLC3A1	Homozy	C.1711 T > A P.571cys571ser			+
18	F	12 years	-	N	N	-	211	350	718	558	1,837					+ (Tio)	
19	F	3 years	+	AbN	AbN	+	211	97	357	224	889	SLC7A9	Homozy	C.1166 C > T P.Thr389Met		+ (Tio)	
20	F	18 months	+	N	N	-	177	235	594	557	1,563	SLC3A1	Homozy	C.1400 T > A P.Met467lys		+ (Tio)	
21	M	10 years	+	++	N	-	112	108	197	244	661	SLC3A1	Homozy	C.1400 T > A P.Met467lys		+ (Pen)	
22	F	10 years	-	N	N	-	93	85	158	177	453	SLC3A1	Homozy	C.1400 T > A P.Met467lys		+ (Pen)	
23	M	Birth	-	N	N	-	98	163	267	337	865	SLC3A1	Homozy	C.1711 T > A P.571cys571ser		+ (Tio)	+

*Biochemical and genetic results for all 23 patients, including urine amino acid (mmol/mmol of creatinine), SLC3A1, and SLC7A9 gene sequencing and del/dup study*.

*N, normal; AbN, abnormal; homozy, homozygous; hetero, heterozygous; Uni, unilateral; bi, bilateral; Pen, penicillamine; Tio, Tiopronin*.

### Genetic Studies

We found thirteen patients who were homozygous for the *SLC3A1* gene, two who were heterozygous for the *SLC3A1* gene, seven who were homozygous for *SLC7A9* gene variants, and one who was compound heterozygous for the *SLC7A9* gene. Five variants were of unestablished pathogenicity: *SLC7A9* homozygous variant C.997_1012del (P.Arg333serfs), *SLC7A9* heterozygous variant C.376g > A (P.Ala126Thr), *SLC7A9* homozygous variant C.460_471del (P.Leu154_Ala157del), *SLC7A9* homozygous variant C.1060G > A (p.A354T), and *SLC3A1* homozygous variant c.1617+1097T > A. Integrated data from the clinical and stone analyses and biochemical profile of the high excretion of dibasic amino acids suggested the pathogenicity of these novel variants. Most of our cohort was homozygous recessive and classified based on genotyping as 13 patients were AA, two patients were A and seven patients classified as BB. All of our patients who inherited two recessive *SLC3A1* mutations from their heterozygous asymptomatic parents and had nephrolithiasis in the first decade of life. Asymptomatic sibling screening revealed either wild type or heterozygous carriers. However, we could not confidently classify our patients based on biochemical classification because we do not have complete biochemical data about the heterozygous, asymptomatic family members. Overall, the most common pathogenic mutations were biallelic missense variants in the *SLC7A9* or *SLC3A1* gene, and one patient had a homozygous duplication encompassing exon 5 to exon 9 of the *SLC3A1* gene (Table 2).

## Discussion

The clinical and genetic characterization, as well as clinical progress of patients with cystinuria from two centers in Saudi Arabia, revealed variable genotypes and phenotypes, and our data did not suggest genotype-phenotype or biochemical and clinical phenotype correlations in these patients. Nephrolithiasis presented in the first decade in more than 90% of our study cohort, which might be explained by the homozygous form of cystinuria. In the previous studies, >80% of patients developed their first stones within the first two decades of life (6, 16). Twenty-one percent of the patients did not pass their first stone until they were >40 years old. In the same studies, the authors highlighted the importance of underlying inherited disease as an etiology of renal stone in older age groups (6).

A mutation in *SLC3A1* results in rBAT protein misfolding and trafficking defects and a mutation in *STC7A9* causes either inactivation of the b(0,+)″ system or trafficking defects. In either case, there is a failure to reabsorb filtered cystine and dibasic amino acids in the proximal tubule of the kidney (2). The loss of the poorly soluble cystine in urine contributes to stone formation. In our patients, homozygous variants in the *SLC3A1* or *STC7A9* genes were the most identifiable underlying genetic causes, but heterozygous and compound heterozygous variants were additional identifiable genetic defects.

Cystine stone formation is the primary symptom of the disease, and it produces significant morbidity in the form of urinary obstruction, and infection. Among cystinuria patients: recurrent urinary tract infections (≥6), staghorn stones, hypertension, and diabetes predicted to be at highest risk for CKD in stone formers patients. However, number of stone attacks, surgical procedures, and symptoms of stone passage were not predicted for CKD (17, 18). Four of our patients developed staghorn calculi as well as recurrent and multiple bilateral renal stones. Nine of the patients in the cohort required thiol therapy at one stage in their lives. Three patients had hypertension later in life, and eight had variable severities of proteinuria. ~47% of the patients had unilateral disease (Tables 1, 2).

Two siblings (patient no. 13 and 15) are heterozygous for a paternal inherited *SLC3A1* variant presented with unilateral stones; one of them had multiple stones only on the right side, leading to nephrectomy of the nonfunctioning kidney. Thereafter, he was free of stones for 14 years on conservative measures only. At present, his brother (patient no. 13) also has right-sided recurrent renal stones, which compels us to think that inherited unilateral disease is linked to a specific genotyping which requires more research in order to study the varying disease patterns. The father is the carrier for this variant and he had never developed stone. This is again supporting that reduced penetrance phenomenon in heterozygous disease-causing mutation. As well as raising the importance of genotyping combined with biochemical profiling as dominant cystinuria patients behave differently with mostly milder and later onset.

However, we have shown that homozygous recessive pattern is the commonest in our population and the *SLC3A*1 gene variant c.1400 T > A (p.Met467Lys) is found in 38% of our cohort. A high rate of consanguinity resulted not only in homozygous disease but also in the presence of the disease in more than one generation in one family.

We have found several affected members in the same family with multiple generations affected. Three families with two or three siblings affected, one family had two generations affected, and 4 (19%) patients had a history of cystinuria in three generations. Patients no. 9, 10, and 14 who are siblings with biallelic mutation c.1400 T > A (p.Met467Lys) at *SLC3A1*gene and no. 13 and 15 siblings are heterozygous for paternal inherited c.1229 A >T variant at *SLC3A1*gene. Patient no. 16 who is homozygous for c.1711T > A (p.571Cys571Ser) variant at *SLC3A1*gene had three adult siblings (not included in the study) with cystine renal stones in addition to the father and paternal aunt. Patient no.17 who is homozygous for c.1711T > A (p.571Cys571Ser) variant at *SLC3A1*gene had affected father and paternal grandmother. Patient no. 19 who is homozygous for c.1166C > T (p.Thr389Met) variant at *SLC7A9* gene has an affected the paternal uncle and the paternal grandmother with cystine stone. Patient no. 20 who is homozygous for c.1400 T > A (p.Met467Lys) variant at *SLC3A1*gene had an affected double cousin, maternal aunt and maternal grandmother. Patient no. 21 is homozygous for c.1400 T > A (p.Met467Lys) variant at *SLC3A1*gene had a maternal uncle and aunt were affected and the maternal grandfather. The presence of multiple generations in the family increase the awareness of the disease presentation as well as initiation of early screening (patient no. 9, 16, 17, and 20).

An intrafamilial phenotypic variations indicates clinical heterogeneity even with similar genotype, which was shown in our study in two families (family 1: patient no. 9, 10, and 14 and family 2: patient no. 13 and 15), in which siblings with the same genetic variation presenting at different ages and with different severities of disease point to a disassociation between the genetic variation and clinical features (6, 7, 19).

Patient no. 1 in the tables has noticed to have isolated cystinuria on several occasions pre and post-treatment and genetic analysis revealed a homozygous *SLC3A1* gene variant c.592del (P.Ala198glnfs^*^8). Brodehl et al. and Eggermann et al. reported the phenotype and the genotype of a family of two with a heterozygous variant T123M at the *SLC7A9* gene resulted in isolated hypercystinuria (20, 21). Patient no. 1 in tables and Eggermann findings indicated that isolated cystinuria is not a separate entity and the presence of isolated renal cystine transport system is unlikely as suggested by Brodehl. However, this variant of the phenotype of isolated hypercystinuria could result either from *SLC7A9* gene *SLC3A1* gene variation and remained unexplained so far.

Although international guidelines and a consensus on the optimal treatment strategy for patients with cystinuria have been established (14, 15, 22, 23). We are following each patient twice /year. We have followed the step-wise treatment approach starting with conservative measures: encourage fluid intake, alkalinizing the urine and dietary modification. If renal stone recurs, we proceeded to pharmacotherapeutic options by adding cystine-binding thiol drugs to the alkalinizing the urine. If still recurs, surgical intervention is required (23). This strategy was applied to all the patients in the current study with variable adherence. Furthermore, current treatments, such as penicillamine (24), often need to be discontinued because of their serious side effect profile, which includes proteinuria. Tiopronin was generally much better tolerated among the current patients than penicillamine, which has also been described by other groups (6, 25, 26). Even with medical therapy, recurrent stone formation is common. We emphasize the importance of understanding the molecular basis of cystinuria and the development of more effective and better-tolerated medications.

Regarding surgical intervention, 78% of patients had received extracorporeal shockwave lithotripsy (ESWL) and/or percutaneous nephrolithotomy (PCNL) due to recurrence while on medical therapy. The preferred procedure for each patient might be different based on the age of the patient; stone characterization including size, surface, number, shape, location; and presence or absence of calcium oxalate coating (2). ESWL is an effective and safe intervention for upper urinary tract stones (27, 28).

However, cystine stones are relatively less susceptible to extracorporeal shock wave lithotripsy (ECWL) than calcium oxalate stones due to the structure of cystine stone and low degree of radiopacity. Therefore, endoscopic surgery, including ureteroscopy and lithotripsy, as well as percutaneous nephrolithotomy (PCNL), are the preferred modalities of stone removal in patients with cystine lithiasis, as they permit irrigation with alkalinizing solution (2, 10, 11).

Dibasic aminoacids excretion rates in obligate heterozygous relatives of cystinuric suffice to give a biochemical classification and the type of cystinuria in the proband which is might be helpful in prognostication and prediction of the disease severity and the risk of renal impairment. One of the limitations in our study was the incomplete data (biochemical profile) of the healthy heterozygous parents and siblings, which would have been influential in the classification of our patient (12, 29, 30).

## Conclusion

Patients with cystinuria in Saudi Arabia often present atypically with early-onset stone formation with a median age of 3 years. In spite of this, renal impairment is very rare; only one patient of our cohort has renal impairment. Despite the high rate of consanguinity, there were wide spectrum of genetic variations. Most of our patients are homozygous recessive with the first stone episode in the first decade of life. There is a weak association of clinical and biochemical profiling with genotype. As such, other factors should be considered to predict the disease course and severity, including environmental factors and different genetic modifiers or epigenetic constitutions.

## Data Availability Statement

The original contributions presented in the study are publicly available. This data can be found here: https://www.ncbi.nlm.nih.gov/clinvar/ with the accession numbers: SUB8123850 SUB8118024 SUB8117883.

## Ethics Statement

The studies involving human participants were reviewed and approved by King Saud University—College of Medicine—Institutional review board Project number E-18-3283. Written informed consent to participate in this study was provided by the participants' legal guardian/next of kin. Written informed consent was obtained from the individual(s), and minor(s)' legal guardian/next of kin, for the publication of any potentially identifiable images or data included in this article.

## Author Contributions

MAlg and KA conceived of the presented idea, developed the theory, and encouraged all the group to investigate further this biochemical defect. MAlg write the final manuscript and draft the figures. MAlf verified the data analysis, methods, and supervised the findings of this work. AT and SS collected the clinical, biochemical, and radiological data, reviewed the literature also helped in drafting the manuscript. MAbd carried out the literature review focusing on pathophysiological defect. RR and JK contributed to the design the study, the analysis of the results, and literature review. MN contributed with clinical details and follow up of adult patients and helped in manuscript. All authors discussed the results and contributed to the final manuscript, provided critical feedback, and helped shape the research, analysis and manuscript. All authors discussed the results and commented on the manuscript.

## Conflict of Interest

The authors declare that the research was conducted in the absence of any commercial or financial relationships that could be construed as a potential conflict of interest.
